# Determining the diagnostic cut-off on the Chinese version of severity of dependence scale for DSM-5 stimulant use disorder

**DOI:** 10.3389/fpsyt.2025.1622306

**Published:** 2025-09-05

**Authors:** Albert Kar Kin Chung, Welton Leung, Cheuk Yin Tse

**Affiliations:** Department of Psychiatry, School of Clinical Medicine, Li Ka Shing Faculty of Medicine, The University of Hong Kong, Hong Kong, Hong Kong SAR, China

**Keywords:** SUD: stimulant use disorder, severity of dependence scale, cocaine, methamphetamine, psychometrics, Chinese-speaking

## Abstract

**Objective:**

To investigate the psychometric properties of the Chinese version of the Severity of Dependence Scale for stimulant (C-SDS-S) in screening for the DSM-5-defined Stimulant Use Disorder (SUD).

**Design:**

Retrospective chart review.

**Methods:**

A total of 227 Chinese-speaking stimulant (methamphetamine and cocaine) users were identified from four previous studies conducted in Hong Kong. Their demographic data, frequency of stimulant use within the past 30 days, scorings for C-SDS-S and the severity of SUD at baseline were extracted and synthesized. In addition, test-retest reliability of C-SDS-S was assessed in 101 subjects who reported C-SDS-S scorings 4 weeks after baseline.

**Findings:**

The C-SDS-S demonstrated an acceptable internal consistency with a Cronbach’s alpha of 0.736. C-SDS-S scorings were associated with the severity of SUD (ρ = 0.292, *p* <.001) and with the frequency of stimulant use within the past 30 days (ρ = 0.196, *p* = .003). All items loaded into one factor which accounted for 50.21% of the variance. Receiver operating characteristic analysis demonstrated that a C-SDS-S cut-off score of ≥ 5 provided optimal discrimination for moderate-to-severe SUD among Chinese-speaking individuals using stimulants. Total scores and individual items of the C-SDS-S demonstrated fair to moderate 30-day test-retest reliability (intraclass correlation coefficient = 0.49; weighted Kappa’s = 0.25-0.46).

**Conclusion:**

The C-SDS-S is a valid and reliable screening instrument to identify stimulant users with DSM-5 defined moderate-to-severe SUD in the Chinese-speaking population.

## 1 Introduction

Amphetamine-type stimulants (ATS) and cocaine are commonly misused drugs globally (1). Not only is ATS the commonest primary drug of misuse in Asia, it also contributes to more than half of its treatment-seeking drug users (1). Among Chinese-speaking countries and regions, including mainland China, Hong Kong, Malaysia, Singapore, and Taiwan, methamphetamine and cocaine account for the highest number of documented cases who come into a formal contact with authorities, more than that of heroin or cannabis (2). Individuals with stimulant use disorder (SUD) are prone to develop a myriad of psychiatric disorders, encompassing mood, bipolar, and psychotic disorders (3–8). Therefore, developing a reliable and valid screening instrument in Chinese for early detection of SUD in Chinese-speaking stimulant users is of paramount importance to administer timely intervention.

The Severity of Dependence Scale (SDS) is a five-item, self-administered scale that measures the psychological dependence of drug users on impaired control, anxiety and preoccupation towards their substance use (9). Rating each item on a Likert scale from 0 to 3 with a maximum score of 15, SDS is designed to contextualize psychological dependence under a dimensional construct with higher scores suggesting more severe dependence. SDS has a single-factor structure with high internal consistency in measuring the construct of substance dependence. It also has a high test-retest reliability and has demonstrated strong associations with the amount and frequency of use across various substances (9–12).

The original English version of SDS had been validated in heroin, cocaine, and amphetamine users (9). Later studies have also translated and validated SDS into various languages, and found different diagnostic cut-offs that allow for the optimal discrimination for substance dependence in accordance with the Diagnostic and Statistical Manual of Mental Disorders (DSM) for alcohol, amphetamines, benzodiazepines, cannabis, cocaine, and heroin (10, 12–20). While the Chinese version of the SDS (C-SDS) has been validated for benzodiazepines, cannabis, heroin, and ketamine (13, 20–22), the psychometric properties of C-SDS for stimulant use have not yet been validated, nor its diagnostic cut-offs for DSM-5 defined SUD established.

The present study aims to establish the diagnostic utility of the C-SDS for stimulant (C-SDS-S) in screening for DSM-5 defined SUD. This study also focuses on determining the cut-off scores that provide optimal discrimination for mild and moderate-to-severe SUD, thus allowing the C-SDS-S to be adopted as a rapid screening instrument in routine clinical practice.

## 2 Methods

### 2.1 Participants

Chinese-speaking stimulant users resided in Hong Kong were retrospectively identified from four previous studies. These studies (UW 18-094, UW 18-095, UW 19–228 and UW 20-189) were approved by the Institutional Review Board of the University of Hong Kong/Hospital Authority Hong Kong West Cluster (HKU/HA HKW IRB). Details of studies UW 18-094, UW 18–095 and UW 20–189 can be reviewed at www.clinicaltrials.gov with study identifiers NCT03485417, NCT03485274 and NCT04373525, respectively. For study UW 19-228, details can be found at www.hkuctr.com with study identifier HKUCTR-2690. In short, these four studies focused on commonly misused drugs in Hong Kong. All the participants included in the present study joined these four studies between August 2018 and October 2023. They were recruited through random sampling from the substance misuse treatment clinics and the community. All these studies were carried out in accordance with the Declaration of Helsinki.

For the current study, we included those participants from these four studies who reported using cocaine and/or methamphetamine for at least six times in the past six months and had completed the C-SDS-S and the Structured Clinical Interview for DSM-5 (SCID-5) on stimulant use disorders at baseline. Cocaine and methamphetamine were pre-selected as the major types of stimulants of concern as these were the two major stimulants of misuse over the past decade in Hong Kong (23). Participants who did not specify the type of stimulants used in their SDS questionnaires, did not use separate SDS questionnaire for different stimulants, had not completed rating on all items, nor completed the SCID-5 assessments would be excluded from the current study.

### 2.2 Design

This was a retrospective data review study. Demographic information, frequency of stimulant use within the past 30 days, scorings on the C-SDS-S, and the severity of SUD verified by board-certified psychiatrists using SCID-5, were retrieved from the study records of participants meeting the inclusion criteria. All participants self-reported their stimulant use over the past 30 days at the time of assessment.

Participants filled in the same C-SDS which was previously translated into traditional Chinese and was validated by Tung et al. (22) and by Chung and Tse (13) in ketamine and cannabis users, respectively. C-SDS-S comprised the same five items from the English version of the SDS: (1) “Did you think your use of (stimulant) was out of control?”; (2) “Did the prospect of missing a smoke/snort make you anxious or worried?”; (3) “Did you worry about your use of the drug?”; (4) “Did you wish you could stop?”; and (5) “How difficult did you find to stop or go without (stimulant)?”. Each item anchored on a 4-point scale. Participants scored the first four items among “0: never/almost never”, “1: sometimes”, “2: often”, and “3: always/nearly always”; while the last item among “0: not difficult”, “1: quite difficult”, “2: very difficult” and “3: impossible”. The total C-SDS-S score ranged from 0 to 15 where a higher score indicates a greater degree of dependence.

This study was approved by HKU/HA HKW IRB (UW 23-267). No external funding sponsored this study.

### 2.3 Statistical analysis

Demographic data, the mean C-SDS-S scores for different severity of SUD, and the frequency of stimulant use in the past 30 days were presented with descriptive statistics. Baseline differences were assessed using one-way ANOVA or the Kruskal-Wallis test for continuous variables, and the Chi-squared test or the Fisher’s exact test for categorical variables. Reliability of the C-SDS-S was assessed by the Cronbach’s alpha. Test-retest reliability of the C-SDS-S was assessed in participants who repeated C-SDS-S scorings 4 weeks after baseline by the intraclass correlation coefficient (ICC) for total scores and Cohen’s weighted Kappa for individual items. Correlations of the scorings on the C-SDS-S with the severity of SUD and the frequency of stimulant use in the past 30 days were assessed for its concurrent validity. Factor analysis using principal component analysis (PCA) was conducted to investigate the construct validity of C-SDS-S. Lastly, receiver operating characteristic (ROC) analysis was performed to determine the area under curve (AUC) and the diagnostic cut-offs on C-SDS-S for DSM-5 defined mild SUD and moderate-to-severe SUD, respectively. Subgroup analyses were performed for cocaine and methamphetamine. All analyses were performed using IBM SPSS Statistics for Windows, Version 29.0, with a significance of alpha = .05.

## 3 Results

### 3.1 Characteristics of participants

Two hundred and twenty-seven participants (36.9%) fulfilling the inclusion criteria were identified from 615 records from the four studies. **Table 1** described their demographics and baseline information of stimulants and other drugs used. Majority of the participants were male (70.9%) and their mean age was 39.8 years old (SD = 10.0). Most of participants (93.8%) had lifetime history of drug use other than stimulants, but less than half of these participants (45.4%) had recent use of other non-stimulant drug within the past 30 days. On average, these poly-drug users had used fewer than one non-stimulant drug in the past 30 days. Eighteen (7.9%) stimulant users did not meet the diagnosis of SUD. A total of 54 users (23.8%) had mild SUD and 155 users (68.3%) had moderate-to-severe SUD. The mean C-SDS-S score for all stimulant users was 5.84 (SD = 3.25), where the average frequency of stimulant use within the past 30 days was 7.7 days (SD = 10.1). A total of 192 participants reported methamphetamine use (84.6%) and 52 participants reported cocaine use (22.9%). Of the 192 methamphetamine users, only one administered methamphetamine via injection with the rest used via smoking. Of the 52 cocaine users, only four used intranasally with powder cocaine and the rest (92.3%) smoked with crack cocaine. Among those methamphetamine users, 13 (6.8%) did not meet the diagnosis of SUD, 52 (27.1%) had mild SUD and 127 (66.1%) had moderate-to-severe SUD; whereas for cocaine users, 8 (15.4%) had no SUD, 9 (17.3%) had mild and 35 (67.3%) had moderate-to-severe SUD. Overall, the age and gender of the participants, their duration of stimulant use, the total number of non-stimulant drug lifetime use and recent use within the past 30 days were similar across different severities of SUD as compared to those with no SUD. Participants with SUD who used methamphetamine had consistently significantly more frequent use than those without SUD (all p’s < 0.05).

**Table 1:** Demographics, C-SDS-S scores, frequency of stimulant use, and substance use history for all participants (N=227).

	No SUD (N=18)	Mild SUD (N=54)	Moderate-to-severe SUD (N=155)
**Age,** mean (range)	41.3 (17-60)	41.0 (23-62)	39.1 (16-60) *Moderate: 39.0 (16-60)* *Severe: 39.3 (17-57)*
Methamphetamine	42.0 (30-60)	40.7 (23-62)	41.7 (20-60) *Moderate: 41.8 (20-60)* *Severe: 41.7 (21-57)*
Cocaine	37.0 (17-57)	40.0 (23-57)	30.4 (16-52) *Moderate: 29.5 (16-42)* *Severe: 31.4 (17-52)*
**Male gender**, N (%)	15 (83.3)	41 (75.9)	105 (67.7) *Moderate: 51 (65.4)* *Severe: 54 (70.1)*
Methamphetamine	12 (92.3)	38 (73.1)	85 (66.9) *Moderate: 40 (64.5)* *Severe: 45 (69.2)*
Cocaine	4 (50.0)	8 (88.9)	25 (71.4) *Moderate: 12 (66.7)* *Severe: 13 (76.5)*
**C-SDS-S scores**, mean (SD)	5.28 (3.83)	4.85 (3.12)	6.25 (3.16) *Moderate: 5.69 (3.03)* *Severe: 6.81 (3.20)*
Methamphetamine	4.77 (3.44)	4.87 (3.15)	5.96 (3.09) *Moderate: 5.40 (2.95)* *Severe: 6.49 (3.14)*
Cocaine	5.87 (4.58)	3.33 (2.74)	7.26 (3.38) *Moderate: 7.00 (3.14)* *Severe: 7.53 (3.69)*
**Frequency of stimulant use in the past 30 days**, mean (SD)	1.83 (3.15)	6.50 (9.13)^*^	8.86 (10.68)^*^ Moderate: 6.79 (9.81)^*^ Severe: 10.96 (11.16)^*^
Methamphetamine	2.15 (3.56)	6.81 (9.36)	9.02 (10.63)^*^ Moderate: 6.61 (9.35)^*^ Severe: 11.32 (11.32)^*^
Cocaine	1.62 (2.20)	5.78 (10.22)	8.29 (10.85) *Moderate: 7.67 (11.31)* *Severe: 8.94 (10.65)*
**Duration of stimulant use in years**, mean (SD)	11.9 (7.7)	9.8 (6.4)	11.3 (8.1) *Moderate: 10.7 (7.9)* *Severe: 11.9 (8.3)*
Methamphetamine	11.4 (7.8)	9.9 (6.5)	11.9 (8.2) *Moderate: 11.1 (7.9)* *Severe: 12.6 (8.5)*
Cocaine	11.9 (7.0)	8.8 (5.6)	7.1 (6.1) *Moderate: 7.2 (5.9)* *Severe: 6.9 (6.5)*
Total number of non-stimulant drug use, mean (SD)			
*Lifetime use*	6.1 (2.7)	5.4 (2.6)	5.9 (3.3) *Moderate: 5.2 (2.7)* *Severe: 6.7 (3.7)*
Methamphetamine	5.9 (3.1)	5.7 (2.9)	6.1 (3.4) *Moderate: 5.3 (2.9)* *Severe: 6.9 (3.7)*
Cocaine	7.3 (2.8)	7.7 (4.2)	6.4 (4.3) *Moderate: 5.1 (2.5)* *Severe: 7.8 (5.3)*
*Recent use within 30 days*	0.61 (0.61)	0.61 (0.68)	0.58 (0.80) *Moderate: 0.50 (0.73)* *Severe: 0.66 (0.85)*
Methamphetamine	0.62 (0.65)	0.67 (0.73)	0.57 (0.77) *Moderate: 0.42 (0.59)* *Severe: 0.72 (0.89)*
Cocaine	0.63 (0.52)	0.89 (0.78)	0.89 (1.05) *Moderate: 0.89 (1.08)* *Severe: 0.88 (1.05)*
**Other non-stimulant drug use**, N (%)			
Lifetime use			
Ecstasy	11 (61.1)	20 (37.0)	84 (54.2) *Moderate: 39 (50.0)* *Severe: 45 (58.4)*
Cannabis	14 (77.8)	45 (83.3)	114 (73.5) *Moderate: 56 (71.8)* *Severe: 58 (75.3)*
Ketamine	13 (72.2)	31 (57.4)	117 (75.5) *Moderate: 54 (69.2)* *Severe: 63 (81.8)*
Heroin	5 (27.8)	17 (31.5)	50 (32.3) *Moderate: 18 (23.1)* *Severe: 32 (41.6)*
Zopiclone	10 (55.6)	22 (40.7)	66 (42.6) *Moderate: 31 (39.7)* *Severe: 35 (45.5)*
Cough medicine	7 (38.9)	22 (40.7)	38 (24.5) *Moderate: 15 (19.2)* *Severe: 23 (29.9)*
Recent use within 30 days			
Ecstasy	0 (0.0)	1 (1.9)	3 (1.9) *Moderate: 1 (1.3)* *Severe: 2 (2.6)*
Cannabis	1 (5.6)	4 (7.4)	25 (16.1) *Moderate: 9 (11.5)* *Severe: 16 (20.8)*
Ketamine	2 (11.1)	2 (3.7)	9 (5.8) *Moderate: 6 (7.7)* *Severe: 3 (3.9)*
Heroin	1 (5.6)	7 (13.0)	21 (13.5) *Moderate: 4 (5.1)* *Severe: 17 (22.1)*
Zopiclone	4 (22.2)	14 (25.9)	26 (16.8) *Moderate: 16 (20.5)* *Severe: 10 (13.0)*
Cough medicine	3 (16.7)	5 (9.3)	6 (3.9) *Moderate: 3 (3.8)* *Severe: 3 (3.9)*

^*^Reference group: no SUD; p < 0.05.

*C-SDS-S*, Chinese version of the Severity of Dependence Scale for stimulant; *SUD*, stimulant use disorder; *N*, number of participants; *SD*, standard deviation.

### 3.2 Validity, reliability, and factor analysis

C-SDS-S demonstrated a weak correlation with the severity of SUD (ρ = 0.292, *p* <.001) and with the frequency of stimulant use within the past 30 days (ρ = 0.196, *p* = .003). Subgroup analyses of the C-SDS-S scorings among methamphetamine users showed similar positive correlations with the severity of SUD (ρ = 0.260, *p* <.001) and the frequency of methamphetamine use (ρ = 0.201, *p* = .005). However, C-SDS-S scorings among cocaine users was only correlated with the severity of SUD (ρ = 0.356, *p* = .011) but not the frequency of cocaine use (ρ = 0.144, *p* = .308). The magnitude of the correlations of C-SDS-S scorings with the degree of severity in SUD suggested that it had better concurrent validity for cocaine than methamphetamine.

The internal consistency of C-SDS-S as measured by the Cronbach’s alpha for all stimulants, methamphetamine alone and cocaine alone were 0.736, 0.738 and 0.744, respectively. Overall, the total score of C-SDS-S has fair test-retest reliability with an ICC of 0.49. Subgroup analyses for methamphetamine and cocaine revealed similar levels of reliability with the corresponding ICCs of 0.52 and 0.50, respectively. For individual items, Item 5 recorded the largest weighted Kappa statistic, followed by Item 2, 4, 3, and 1. Subgroup analyses revealed identical trends among methamphetamine users but not cocaine users, where Item 1 recorded the highest and Item 2 the lowest weighted Kappa statistic (**Table 2**).

**Table 2:** Test-retest reliability (Cohen’s weighted Kappa) of each item on the C-SDS-S.

Item	Stimulants	Methamphetamine	Cocaine
1. Did you think your use of stimulants was out of control?	0.264	0.250	0.385
2. Did the prospect of missing a smoke of stimulants make you anxious or worried?	0.387	0.409	0.267
3. Did you worry about your use of stimulants?	0.315	0.335	0.300
4. Did you wish you could stop?	0.345	0.365	0.347
5. How difficult did you find it to stop or go without stimulants?	0.454	0.463	0.354

*C-SDS-S*, Chinese version of the Severity of Dependence Scale for stimulant.

Regarding factor analysis, the Kaiser-Meyer-Olkin measures of sampling adequacy for all stimulants, methamphetamine alone and cocaine alone were 0.743, 0.754, and 0.727, respectively. PCA identified one factor for stimulants and for the methamphetamine subgroup, but two factors for the cocaine subgroup. Factor 1 explained roughly 50% of the variance in all stimulant users and methamphetamine users, while Factors 1 and 2 accounted for over 70% of the variance in cocaine users (**Table 3**). Most items possessed strong factor loading characteristics (> 0.6) except for Item 4 that had weak factor loadings on Factor 1 (0.38-0.55) (**Table 4**). The C-SDS-S would be more consistent if Item 4 was removed, resulting in Cronbach’s alpha of 0.756, 0.747, and 0.797 for all stimulants, methamphetamine alone and cocaine alone, respectively.

**Table 3:** Principal component analysis of C-SDS-S.

	Stimulants		Methamphetamine		Cocaine	
Factor	Eigenvalues	% of Variance explained	Eigenvalues	% of Variance explained	Eigenvalues	% of Variance explained
1	2.51	50.21	2.53	50.57	2.61	52.18
2	0.99	19.74	0.94	18.78	1.09	21.72
3	0.60	12.09	0.63	12.60	0.59	11.70
4	0.48	9.66	0.48	9.58	0.39	7.78
5	0.42	8.31	0.42	8.48	0.33	6.62

*C-SDS-S*, Chinese version of the Severity of Dependence Scale for stimulant.

**Table 4:** Factor loadings for each item in C-SDS-S.

Item	Stimulants	Methamphetamine	Cocaine	
			Factor 1	Factor 2
1. Did you think your use of stimulants was out of control?	0.76	0.74	0.86	-0.18
2. Did the prospect of missing a smoke of stimulants make you anxious or worried?	0.77	0.80	0.73	-0.45
3. Did you worry about your use of stimulants?	0.77	0.76	0.80	0.28
4. Did you wish you could stop?	0.49	0.55	0.38	0.87
5. How difficult did you find it to stop or go without stimulants?	0.70	0.69	0.74	–

*C-SDS-S*, Chinese version of the Severity of Dependence Scale for stimulant.

### 3.3 ROC analysis

**Figure 1:**
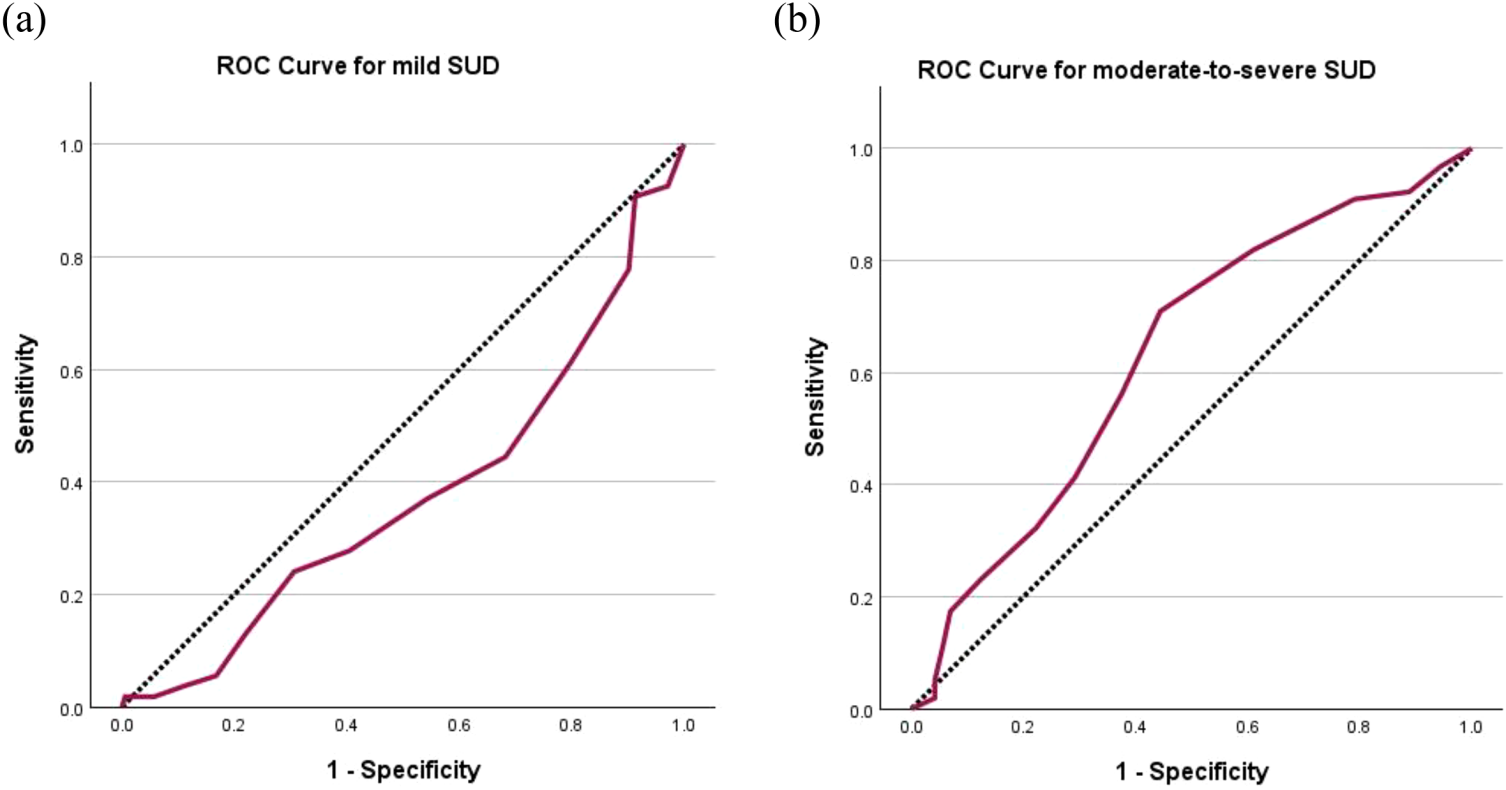
Receiver operating characteristic (ROC) curves of C-SDS-S for **(a)** mild SUD with area under curve (AUC) = 0.377 and **(b)** moderate-to-severe SUD with AUC = 0.632. The diagonal segments were produced by ties. *C-SDS-S*, Chinese version of the Severity of Dependence Scale for stimulant; *SUD*, stimulant use disorder.

The AUC for users with mild SUD was 0.377 (95% CI 0.290 to 0.464), whereas for users with moderate-to-severe SUD was 0.632 (95% CI 0.552 to 0.711) (**Figure 1**). The cut-off score of ≥ 5 yielded the highest Youden Index of 0.27 with a sensitivity of 71.0% and a specificity of 55.6% (**Table 5**). It follows that the C-SDS-S had the greatest diagnostic utility for moderate-to-severe SUD but not mild SUD, and stimulant users scoring 5 points or higher on the C-SDS-S would likely be suffering from a moderate-to-severe SUD.

**Table 5:** Criterion validity of C-SDS-S at each successive cut-off score on the optimal discrimination for DSM-5 moderate-to-severe SUD in stimulant users (N=155).

Stimulants				Methamphetamine			Cocaine		
Score	Sensitivity	Specificity	Youden Index	Sensitivity	Specificity	Youden Index	Sensitivity	Specificity	Youden Index
≥ 1	96.8	5.6	0.02	96.1	6.2	0.02	97.1	17.6	0.15
≥ 2	92.3	11.1	0.03	91.3	10.8	0.02	94.3	29.4	0.24
≥ 3	91.0	20.8	0.12	89.0	21.5	0.11	–	–	–
≥ 4	81.9	38.9	0.21	79.5	40.0	0.20	88.6	47.1	0.36
≥ 5	71.0	55.6	0.27	66.9	56.9	0.24	85.7	52.9	0.39
≥ 6	56.1	62.5	0.19	53.5	63.1	0.17	65.7	58.8	0.25
≥ 7	41.3	70.8	0.12	39.4	70.8	0.10	48.6	76.5	0.25
≥ 8	32.3	77.8	0.10	29.9	80.0	0.10	42.9	76.5	0.19
≥ 9	23.2	87.5	0.11	20.5	87.7	0.08	37.1	88.2	0.25
≥ 10	17.4	93.1	0.11	15.0	93.8	0.09	28.6	94.1	0.23
≥ 11	11.0	94.4	0.05	7.9	95.4	0.03	22.9	94.1	0.17
≥ 12	5.2	95.8	0.01	3.1	96.9	0.00	11.4	94.1	0.06
≥ 13	2.6	95.8	-0.02	1.6	96.9	-0.02	5.7	94.1	0.00
≥ 14	1.9	95.8	-0.02	–	–	–	2.9	94.1	-0.03

*C-SDS-S*, Chinese version of the Severity of Dependence Scale for stimulant; *SUD*, stimulant use disorder; *N*, number of participants.

**Figure 2:**
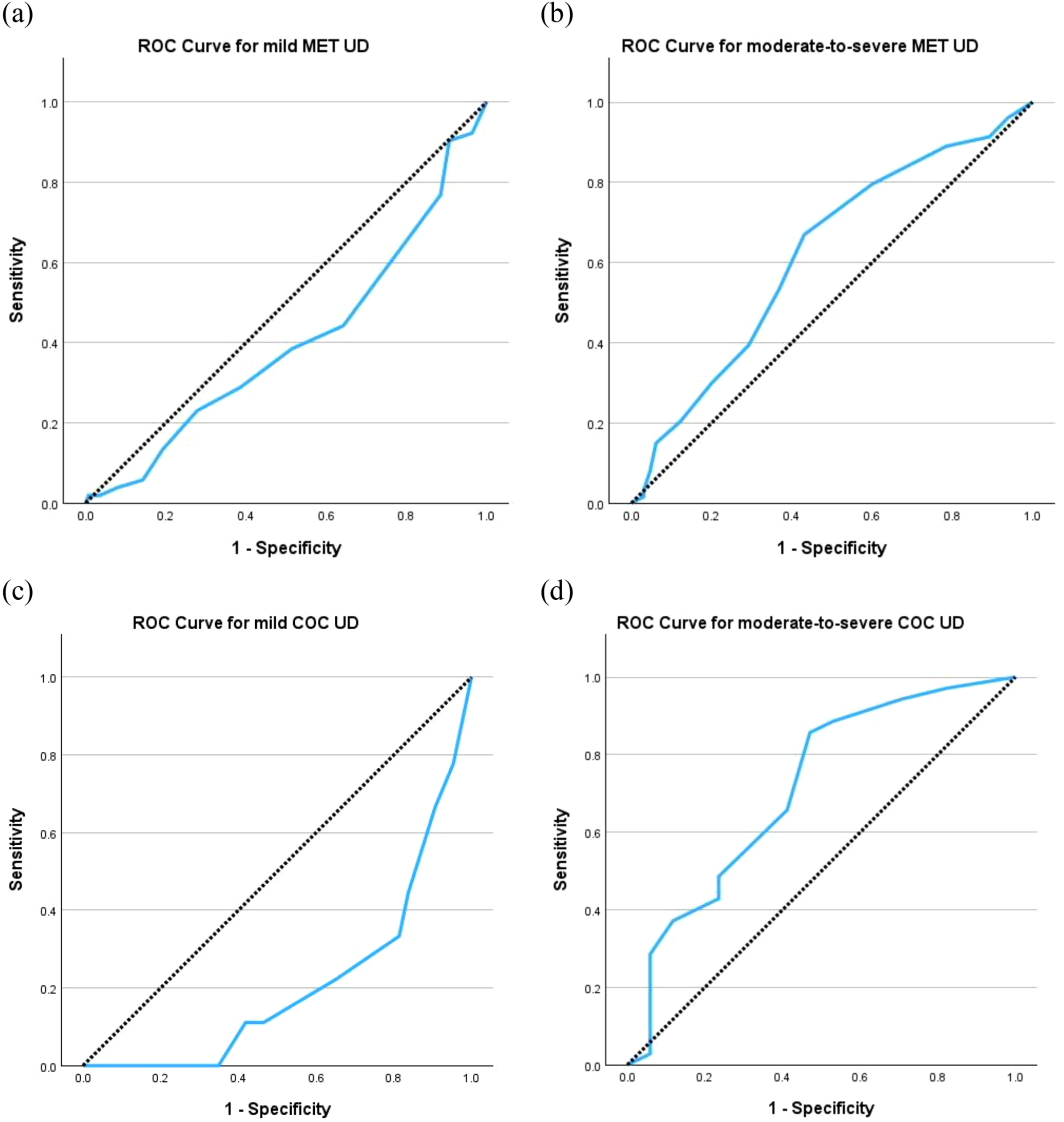
Receiver operating characteristic (ROC) curves of C-SDS-S for **(a)** mild UD for methamphetamine (MET) with area under curve (AUC) = 0.401, **(b)** moderate-to-severe UD for MET with AUC = 0.618, **(c)** mild UD for cocaine (COC) with AUC = 0.208, and **(d)** moderate-to-severe UD for COC with AUC = 0.712. The diagonal segments were produced by ties. *C-SDS-S*, Chinese version of the Severity of Dependence Scale for stimulant; *UD*, use disorder.

Subgroup analyses demonstrated low AUCs for both mild SUD of methamphetamine (0.401, 95% CI 0.309 to 0.493) and cocaine (0.208, 95% CI 0.060 to 0.356), but those for moderate-to-severe SUD of methamphetamine and cocaine were as high as 0.618 (95% CI 0.533 to 0.703) and 0.712 (95% CI 0.555 to 0.869), respectively (**Figure 2**). The same cut-off scores of ≥ 5 identified moderate-to-severe SUD for both methamphetamine (sensitivity = 66.9%, specificity = 56.9%, Youden Index = 0.24) and cocaine (sensitivity = 85.7%, specificity = 52.9%, Youden Index = 0.39) (**Table 5**).

## 4 Discussion

The present study explored the validity and reliability of the C-SDS-S as a clinical tool to screen for mild and moderate-to-severe DSM-5 SUD in Chinese-speaking stimulant users. We showed that a higher total score on the C-SDS-S is associated with greater SUD severity and the frequency of stimulant use, especially among methamphetamine users. Overall, a single-factor structure was observed for the C-SDS-S and when used among methamphetamine users, consistent with other studies that demonstrated a one-dimensional structure of the SDS when administered in different languages and for various substances (9, 12, 13, 16, 17, 21, 22, 24, 25). The two-factor structure identified among our cocaine samples who are predominantly crack cocaine users corroborated with the previous study by Ferri et al. (24). Overall, our findings suggest that C-SDS-S may serve as a preliminary screening instrument in selected clinical contexts, particularly for moderate-to-severe stimulant use disorders, although its diagnostic performance remains limited.

Our study also established a cut-off score of five for DSM-5 defined moderate-to-severe SUD, regardless if the stimulant used was methamphetamine or cocaine. These cut-off scores were both higher than those established in Australia and Spain two decades ago, which were four for methamphetamine and three or four for cocaine (15, 19). It follows that the diagnostic utility of SDS is highly contextual. For the same substance, the diagnostic cut-off for the optimal discrimination of SDS-defined dependence might be gender- and ethnic-specific, in which the difference could be as large as 2 points between male and female, and between Westerners and non-Westerners (12). Cabana-Dominguez et al. revealed that cocaine dependence is a highly heritable disorder within the European ancestry, though little is known among the Asian-ancestry cocaine users (26). On the other hand, recent genome wide association studies demonstrated the significant loci for methamphetamine dependence might be different between the European-, African- and Chinese- ancestries (27, 28). Such genetic variabilities could result in different single nucleotide polymorphism-based expression on the nicotinic acetylcholine receptor activity at the reward circuitry, axonal pruning in hippocampus, and might potentially lead to diverse trajectories in the development of methamphetamine use disorder. These variations suggest that different SDS cut-off scores may be necessary, in particular for different population across ancestries. We did not perform subgroup analyses for male and female participants due to the small number of female participants.

In addition, changes in the diagnostic criteria for substance use disorder might have attributed to the disparity in diagnostic cut-offs across multiple substances. In most validation studies, the 4^th^ edition of the DSM (DSM-IV) was adopted as the “gold standard”. According to DSM-IV, substance abuse and dependence were independently categorized using two separate sets of diagnostic criteria. Under DSM-5, however, substance use disorder was defined using a set of diagnostic criteria primarily adopted from that of DSM-IV abuse and DSM-IV dependence (29). The change from a dichotomous to a continuous diagnostic construct warrants the validation of the C-SDS-S against the DSM-5 criteria to ascertain its criterion validity. Also, similar to our previous work in cannabis (13), we established the diagnostic cut-off on C-SDS for moderate-to-severe SUD but not for mild SUD (13). While the original SDS was developed with a single factor structure under a unidimensional construct for dependence, such single-factor solution might not hold equally valid for non-dependent low-level drug users (30, 31). Therefore, its diagnostic utility in detecting milder states as in abuse or harmful use might be low. Given the clinical significance of timely identification of mild SUD for early intervention, the generalizability of the C-SDS-S in detecting mild SUD warrants further investigation.

While most validation studies on SDS to date identified a unidimensional structure, PCA revealed a two-factor structure for cocaine with Item 4 contributing to the second factor. This is consistent with the validation study of C-SDS in benzodiazepines in Taiwan (20), which also showed the scorings on Item 4 were similar between dependent and non-dependent users. Factor loading for Item 4 is consistently and substantially lower than all other items of the C-SDS-S in the present study, and the internal consistency of the C-SDS-S would improve if Item 4 was removed. Although repeated attempts to quit or reduce stimulant use is one of the diagnostic criteria for DSM-5 SUD, the lower factor loadings of Item 4 suggested that those with stimulant dependence might not have adequate motivation to alter their drug use behavior. Stimulant-dependent users might also hesitate in the prospect of withdrawal symptoms, hence are ambivalent to quit or reduce stimulant use despite its myriad of consequences. In fact, repeated exposure to stimulants reinforces the negative state manifested as craving, withdrawal symptoms, and/or emotional distress (32). This upregulated negative state following prolonged stimulant exposure in dependent users might supersede their desire to quit or reduce stimulant use. Besides, cyclothymic and irritable temperaments have been associated with cocaine and stimulant abuse and heavy uses (33). These temperamental predispositions in stimulant users may heighten their vulnerability to emotional dysregulation during such negative state and contribute to their motivational ambivalence, as reflected by a lower factor loading for Item 4 (“wish to stop”) in the C-SDS-S.

One of the limitations of the present study is the small sample size of cocaine users, which could have attributed to the disparity between the results from the subgroup analyses on cocaine and existing literature, as well as the methamphetamine subgroup and the overall model in the present study. For instance, Item 1 had the lowest test-retest reliability in methamphetamine users and the entire cohort, but was the highest in cocaine users. Among the 101 participants who had repeated C-SDS-S scorings within four weeks, only 16 were cocaine users. Such sample imbalance in particular for the cocaine subgroup may also affect the ROC analysis and the subsequent skewed AUC. All these can inevitably undermine the certainty of the results. As opposed to previous validation studies (9, 11), the small number of cocaine users in the present study might also explain the independence between C-SDS-S scorings and frequency of use within this subgroup where no statistical difference was observed despite showing patterns similar to the methamphetamine subgroup. An early epidemiological study in the United States found that the number of days of stimulant use over the past 12 months was higher in dependent users than those with abuse (34). A different relationship between C-SDS-S scores and frequency of use in cocaine users might be observed if more users could be included. Furthermore, the unique pharmacodynamics of cocaine as a predominant dopamine-reuptake inhibitors as opposed to methamphetamine as a dopamine “releaser”, along with their differing pharmacokinetics, may lead to the distinct use pattern and dependence model specific to cocaine. All these suggest that a single version of C-SDS-S might not fit all stimulant types equally well.

Another limitation of our study is that we were only able to access data in a single locality. Whether the results can be generalized to other Chinese-speaking communities warrants further investigation. Moreover, the cross-sectional design of this study limits the predictive validity of using the C-SDS-S. In addition, our study only assessed the use disorders of methamphetamine and cocaine, both being the predominantly misused stimulants in our locality. Further investigations targeting novel stimulants and psychoactive substances such as khat is warranted.

## 5 Conclusion

The present study supports the application of the C-SDS-S as a valid and reliable screening instrument for moderate-to-severe SUD in Chinese-speaking stimulant users. A cut-off score of 5 provides optimal discrimination for both methamphetamine and cocaine and should prompt further diagnostic assessments by broad-certified psychiatrists. Additionally, revising Item 4 of the C-SDS-S may enhance its accuracy for screening purposes in Chinese-speaking stimulant users. Notwithstanding its high validity and reliability, we urge the validation of the SDS in accordance with the updated diagnostic criteria defined in DSM-5 to maximize its diagnostic utility in screening for both mild and moderate-to-severe substance use disorders in routine clinical settings.

## Data Availability

The original contributions presented in the study are included in the article/supplementary material, further inquiries can be directed to the corresponding author/s.
